# Effect of the Frequency of Rehabilitation Treatments on the Long-Term Mortality of Stroke Survivors with Mild-to-Moderate Disabilities under the Korean National Health Insurance Service System

**DOI:** 10.3390/healthcare11111587

**Published:** 2023-05-29

**Authors:** Dougho Park, Kang Ju Son, Jong Hun Kim, Hyoung Seop Kim

**Affiliations:** 1 Department of Rehabilitation Medicine, Pohang Stroke and Spine Hospital, Pohang 37659, Republic of Korea; 2 Department of Medical Science and Engineering, School of Convergence Science and Technology, Pohang University of Science and Technology, Pohang 37673, Republic of Korea; 3 Department of Research and Analysis, National Health Insurance Service Ilsan Hospital, Goyang 10444, Republic of Korea; 4 Department of Neurology, National Health Insurance Service Ilsan Hospital, Goyang 10444, Republic of Korea; 5 Department of Physical Medicine and Rehabilitation, National Health Insurance Service Ilsan Hospital, Goyang 10444, Republic of Korea

**Keywords:** stroke, rehabilitation, national health insurance, long-term care, mortality

## Abstract

Given the increase in stroke-related social costs, studies on survival and functional prognosis after stroke are urgently needed. Therefore, we investigated the relationship between the frequency of rehabilitation treatments in the acute and subacute phases of stroke and the long-term mortality of stroke survivors with mild-to-moderate disabilities. We performed a retrospective cohort study using data from the Korean National Health Insurance Service database. Our final cohort included 733 patients with national disability registration grades 4–6. The number of special rehabilitation treatment claim codes was used as a proxy for the frequency of rehabilitation treatments. Furthermore, we categorized the rehabilitation frequencies within 24 months of stroke onset as 1–50, 51–200, 201–400, and >400. The dependent variable was all-cause mortality, and it was evaluated from 24 to 84 months after stroke onset. Severe disability was associated with a lower long-term mortality rate in the chronic phase (*p* < 0.001). In the Cox regression analysis, severe disability, older age, male sex, and chronic kidney disease were independent risk factors for long-term mortality in patients with stroke and mild-to-moderate disabilities. However, the frequency of acute/subacute rehabilitation treatments did not significantly improve long-term mortality. Our results suggest that the association between rehabilitation frequency and lower long-term mortality for patients with mild-to-moderate stroke was inconclusive. Therefore, further study is needed to determine a better-customized rehabilitation treatment system for these patients.

## 1. Introduction

Presently, South Korea has a rapidly aging population. As of 2020, the proportion of those aged ≥65 years was 16.4% [1], and the prevalence of geriatric cerebral diseases, such as stroke, has increased [2]. Since the social and economic burdens of stroke are expected to increase continuously due to its high morbidity [3,4], the following are urgently needed: (1) analysis of the long-term prognosis of stroke survivors and (2) application of relevant results for efficient allocation of medical resources, as well as establishment of a proper medical delivery system for patients with stroke.

Even though the mortality rate after stroke has declined due to recent advances in acute treatment [5], many survivors still suffer from functional dependency [6,7]. Therefore, post-stroke rehabilitation is crucial for short-term and long-term prognoses. Studies have shown that intensive post-stroke rehabilitation reduces disability and improves activities of daily living (ADLs) in stroke survivors [8,9]. In particular, Yagi et al. [10] reported that early and intensive rehabilitation improved ADL scores in patients with ischemic stroke in a nationwide study of 100,719 patients. Furthermore, the degree of post-stroke rehabilitation favors long-term survival [11,12]. For instance, in Taiwan, Chang et al. [13] followed-up on patients with 4594 first-ever strokes for 32 months and reported that rehabilitation reduced the risk of readmission and death. Thus, these previous studies have demonstrated the importance of acute rehabilitation after stroke.

The Korean National Health Insurance Service (NHIS) has a policy that generally covers essential rehabilitation for up to 2 years after stroke onset. However, due to the conventional claim acceptance criteria of post-stroke rehabilitation by the Health Insurance Review and Assessment (HIRA) service, some patients with stroke largely depend on inpatient treatment regardless of the severity of the stroke [14,15]. In addition, NHIS-covered rehabilitations are accessible at relatively low prices for a certain period, coupled with the scarcity of community-based rehabilitation programs [16]. Furthermore, the focus of the HIRA service’s rehabilitation treatment coverage criteria on the passage of time might have instigated this phenomenon again. Moreover, there has been a lack of optimal guidelines or recommendations for stroke rehabilitation delivery systems [17,18]. Therefore, it is necessary to verify whether the high frequency of rehabilitation treatments in the acute/subacute phase favors the prognosis of the chronic stage in stroke survivors while reflecting on the characteristics of the Korean healthcare system using the NHIS database.

In this study, we aimed to investigate whether the findings from previous studies can be extrapolated to stroke survivors with mild-to-moderate disabilities. In addition, we sought to determine if the higher frequency of rehabilitation treatments during the acute and subacute phases, which has been found to independently reduce the risk of long-term mortality in stroke survivors with severe functional limitations under the Korean NHIS system [19], also holds for individuals with milder-to-moderate impairments. Therefore, to address these challenges, we utilized the comprehensive nationwide cohort dataset provided by the Korean NHIS and analyzed the impact of rehabilitation intensity during the acute and subacute phases on the long-term mortality of stroke survivors, specifically in those within the mild-to-moderate disability range.

## 2. Materials and Methods

### 2.1. Data Source and Study Design

We conducted a longitudinal study using a nationwide population cohort database from the Korean NHIS. This study was approved by the Institutional Review Board of NHIS Ilsan Hospital (approval number: 2021-07-042). Given the retrospective study design and the anonymity of the NHIS data, the requirement for informed consent was waived. This study was also conducted in accordance with the principles of the Declaration of Helsinki.

**Figure 1:**
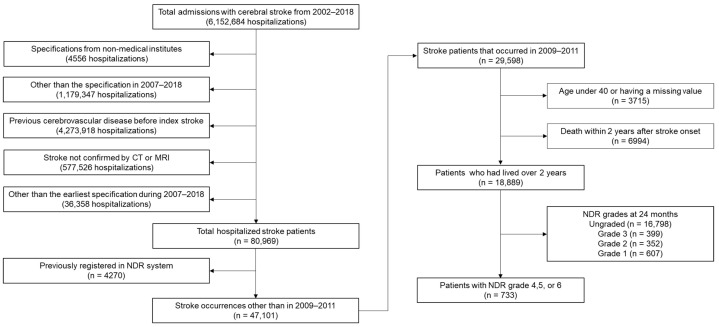
Flowchart of patient inclusion in this study. Abbreviation: NDR, national disability registration.

**Figure 2:**
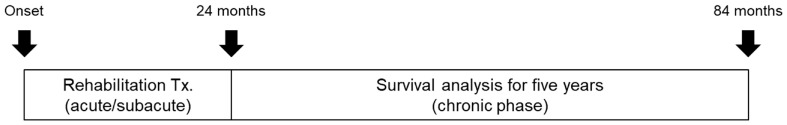
Two-year landmark method for Cox regression analysis. Abbreviation: Tx., treatments.

The study cohort comprised 6,152,684 hospitalized patients diagnosed with stroke from 2002 to 2018, and the International Classification of Diseases (ICD-10) codes used to diagnose stroke included I60, I61, I62, I63, and I64. Among the study cohort, 29,598 patients with their first-ever stroke between 2009 and 2011 were selected. For the final dataset, we applied the following exclusion criteria: (1) age ≥40 years, (2) missing data, (3) death within 2 years of stroke onset, and (4) national disability registration (NDR) grades below 4 within 24 months of onset. Eventually, we included 733 patients who survived at least 2 years after stroke and had NDR grades of 4–6 (Figure 1). Detailed definitions of the eligible NDR grades for this study are presented in Table 1. Our endpoint was all-cause mortality. We analyzed long-term survival of 5 years from 24 to 84 months after stroke onset (Figure 2).

### 2.2. Covariates

Age groups were categorized by decade. Furthermore, we defined patients’ socioeconomic status based on their insurance premium levels. The levels were categorized as medical aid and quartiles of the national health insurance premium levels (Table S1). The patients’ residential areas were defined as capital, metropolitan, city, and county. In addition, hypertension, diabetes, dyslipidemia, ischemic heart disease, atrial flutter/fibrillation, and chronic kidney disease (CKD) were categorized as co-morbidities. Co-morbidities were identified based on the corresponding ICD-10 codes (Table S2). In addition, the stroke subtypes were categorized as subarachnoid hemorrhage, intracranial hemorrhage, ischemia, or unspecified. Finally, the frequency of acute and subacute rehabilitation treatments was defined as the number of special rehabilitation codes (MM105) billed to the HIRA service within 24 months of onset.

We used the Korean NDR system of cerebral lesions for disabilities. All patients with stroke could apply for NDR after adequate treatment for at least 6 months of onset. Grading according to the NDR system was performed by a specialist in rehabilitation medicine, neurosurgery, or neurology and was primarily based on physical examination and the modified Barthel index (MBI). Furthermore, medical records containing the patient’s post-stroke treatment history and brain imaging findings were used as grading criteria. We set grades 4–6 in the NDR system as mild-to-moderate disabilities. In Grade 4, patients could walk and perform most ADLs with occasional need for help from others; this grade corresponds to an MBI score of 70–80. Grade 5 patients could walk and perform most ADLs independently, but often not perfectly; this grade corresponds to an MBI score of 81–89. Finally, grade 6 patients could walk and perform most ADLs independently; however, performance time was occasionally slow, or behavior was abnormal. This grade corresponds to an MBI score of 90–96 (Table 1).

### 2.3. Statistical Analysis

The chi-squared (trend) test was applied for comparisons between the defined groups. Kaplan–Meier curves and log-rank tests were used for cumulative survival analysis. The Cox regression analysis was used to establish a Cox-proportional hazards model to identify independent risk factors for long-term survival in our final cohort. Furthermore, we established stratified Cox-proportional hazards models to exclude interactions between NDR grade and the rehabilitation frequencies. In addition, we applied the 2-year landmark Cox regression method for treating a time-varying feature of rehabilitation treatments frequency and NDR grade. All statistical analyses were performed with SAS version 9.4 (SAS Institute, Inc., Cary, NC, USA). A value of *p* < 0.05 was considered to be statistically significant.

## 3. Results

### 3.1. Baseline Features

In our study cohort, 254 patients received no rehabilitation within 24 months of onset, 209 received 1–50, 138 received 51–200, 76 received 201–400, and 56 received >400. Comparative analyses revealed that the frequency of rehabilitation treatments was higher in urban areas, and the ratio of patients receiving no rehabilitation was higher at the county level (*p* = 0.037). In addition, the lower the NDR grade (i.e., the more severe the disability), the higher the frequency of rehabilitation treatments (*p* < 0.001). No significant differences were observed between the groups regarding age, sex, socioeconomic status, and co-morbidities (Table 2).

The NDR grades included 166 grade 6, 215 grade 5, and 352 grade 4 patients. The lower the NDR grade, the higher the age (*p* = 0.009) and frequency of rehabilitation treatments (*p* < 0.001). No other variables showed significant differences between the NDR grade groups (Table S3).

### 3.2. Long-Term Mortality and Cox-Proportional Hazards Models

**Figure 3:**
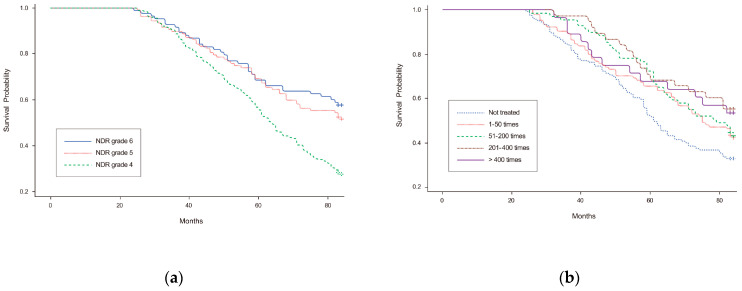
Cumulative survival rates in patients with stroke and mild-to-moderate disabilities. (**a**) The groups with severe disabilities showed a lower chronic-phase survival rate. (**b**) Higher frequencies of rehabilitation in the acute/subacute phases did not show significantly higher chronic-phase survival rate. Abbreviation: NDR, national disability registration.

In the cumulative survival analysis, NDR grade 4 showed a significantly lower survival rate than grades 5 and 6 (*p* < 0.001) (Figure 3a). However, no significant difference was observed in the long-term survival rates between the groups regarding the frequency of rehabilitation treatments (*p* = 0.215) (Figure 3b).

In the Cox-proportional hazards model, a low frequency of acute/subacute rehabilitation treatments did not increase long-term mortality risk; rather, old age was identified as an independent risk factor. The highest risk was observed in those aged >80 years (adjusted hazard ratio (aHR), 11.40; 95% confidence interval (CI), 3.74–34.77; *p* < 0.003), followed by those in the seventh (aHR, 3.15; 95% CI, 1.08– 9.17; *p* = 0.036) and eighth decade (aHR, 5.80; 95% CI, 2.01–16.76; *p* = 0.001) groups. Notably, other variables observed as independent risk factors for long-term mortality of stroke survivors were NDR grade 4 (aHR, 1.82; 95% CI, 1.23–2.68; *p* = 0.003), male sex (aHR, 1.82; 95% CI, 1.36–2.44; *p* < 0.001), and CKD co-morbidity (aHR, 2.85; 95% CI, 1.17–6.96; *p* = 0.021) (Table 3).

In patients with NDR grade 4, mild-to-moderate rehabilitation frequencies significantly reduced chronic-phase mortality (51–200 rehabilitations: aHR, 0.46; 95% CI, 0.27–0.76; *p* = 0.005 and 201–400 rehabilitations: aHR, 0.50; 95% CI, 0.26–0.97; *p* = 0.040), whereas rehabilitation frequencies of >400 showed no significant reduction in the risk of chronic-phase mortality. In contrast, in patients with NDR grade 6, the rehabilitation frequencies of >400 showed a significantly higher risk of chronic-phase mortality (aHR, 15.00; 95% CI, 2.03–111.10; *p* = 0.008) (Table 4).

## 4. Discussion

One of the key findings of this study is that the frequency of acute/subacute rehabilitation treatments was not significantly associated with the long-term mortality risk of stroke survivors with mild-to-moderate disabilities. However, well-known risk factors such as age, male sex, severe disability, and CKD were identified as risk factors for mortality in stroke survivors with mild-to-moderate disabilities [20]. In addition, in the stratified analyses to determine the interactions between disabilities and rehabilitation frequencies, we not only confirmed that in some patients with moderate disabilities (NDR grade 4), acute/subacute rehabilitation independently lowered the risk of chronic mortality, but also that a high frequency of rehabilitation treatments was an independent risk factor for chronic mortality in stroke survivors with mild disabilities (NDR grade 6). Thus, we consider that the higher frequency of rehabilitation treatments in mild cases was probably related to the extended length of hospital stays due to secondary medical conditions or complications after the stroke event rather than the rehabilitation treatment itself, which increased the mortality risk. Importantly, it is worth noting that our results were inconclusive in explaining the rehabilitation frequency and lower long-term mortality for patients with mild-to-moderate stroke, since we have demonstrated the need for an effective rehabilitation medical delivery system and guidelines for appropriate rehabilitation instead of providing novel universal rehabilitation treatment after stroke for these patients, considering the limited available medical resources. Nevertheless, we believe our study can serve as a basis for policy considerations regarding long-term care for stroke survivors.

The strength of our study is that it was conducted using a nationwide database. South Korea currently administers the NHIS [21], and we objectively obtained data on the number of rehabilitations based on the claims data provided to the HIRA service. Regarding stroke, major rehabilitation items, including special rehabilitations for 2 years after stroke onset, are NHIS-covered and universally provided at low prices. Such wide coverage at affordable prices for rehabilitation was the principal motivation behind our study design, in which we investigated the relationship between rehabilitation treatment frequency over 2 years and the long-term survival rate of patients with stroke in the chronic phase. Particularly, in this study, we presumed that the number of prescriptions for special rehabilitation could be used to estimate the total amount of rehabilitation because special rehabilitation, the target variable used in the present study, is prescribed by a rehabilitation specialist and is the basic prescription code for rehabilitation due to central nervous system lesions.

However, our results differed from those of previous studies. Hsieh et al. [22] reported that high-intensity rehabilitation within the first 90 days lowered the mortality risk among patients with mild-to-moderate stroke. Similarly, Cheng et al. [23] also reported that the risk of recurrent stroke and mortality was significantly lower with increased frequency of rehabilitation treatments within 1 year after stroke in patients with a Charlson co-morbidity index of <3. Therefore, we consider that the discrepancies between our results and those of the abovementioned studies may be due to (1) differences in the definition of disease severity, (2) differences in the follow-up period for survival analysis, and (3) differences in rehabilitation delivery systems [24]. Furthermore, we performed survival analysis for the period ranging from 2 to 7 years after the onset of stroke to focus on stroke survival in the chronic phase, since studies have shown that in the acute phase of stroke, patients’ survival rate was largely dependent on the characteristics and severity of the stroke and its complications rather than the extent of rehabilitation [25,26]. Therefore, we aimed to minimize this bias in this study and observed how 2 years of NHIS-covered rehabilitation impacted the long-term mortality of stroke survivors. We believe that our unique study design was probably why our results differ from those of previous studies.

In this study, subgroup targeting was used to analyze the relationship between rehabilitation intensity and long-term mortality because stroke is heterogeneous according to its type, mechanism, location, and size of lesion [27]. Several stroke-related studies have also emphasized the importance of subgroup targeting, as through subgroup targeting, more reliable results can be obtained by reducing intersubject variables [28,29]. We used the NDR grades registered in the NHIS for subgrouping, with the assumption that disability grades reflect stroke severity. We believe the subgrouping analysis we performed is another strength of this study because we utilized objective criteria based on national data, as carried out earlier when obtaining data for the frequency of rehabilitation treatments.

As another subgrouping method, we aimed to analyze the long-term survival patterns of typical adult patients with stroke in South Korea. Although stroke is uncommon among those of lesser age, young-age stroke frequently accompanies congenital abnormalities such as Moyamoya disease or arteriovenous malformation [30,31]. In such cases, we speculated that the underlying diseases would significantly affect the survival rates of patients in the chronic phase. Therefore, we limited our study cohort to patients aged >40 years to minimize such bias.

Consequently, we believe our results can be used as a basis for long-term treatment plans for stroke survivors needing post-stroke rehabilitation. Moreover, given the limited healthcare resources in South Korea, this study can be used for the efficient allocation of medical resources according to severity. The prescription of NHIS coverage for post-stroke rehabilitation by rehabilitation specialists frequently focuses on the period after stroke onset. Such focus is based on the theoretical assumption that functional recovery after stroke follows a logarithmic pattern and that most recovery occurs in the acute and early subacute phases [32,33]. However, the disadvantage of such a system is that some mild-stroke patients can unnecessarily extend the length of their hospital stay even when they do not require further hospitalization [34]. Likewise, some severe patients requiring functional maintenance therapy may not receive adequate maintenance treatment because the duration of their stroke has exceeded 2 years. Since our previous study showed that a higher frequency of rehabilitation treatments for up to 24 months after stroke onset lowered the long-term survival of 593 patients with stroke and severe disabilities (NDR grade ≤ 3) [19], we suggest that the results of the present study do not negate the need for early and intensive rehabilitation in the acute and subacute phases. Instead, we found that patients with NDR grade 4 had a decreased chronic-phase mortality with a moderate frequency of rehabilitation treatments. Therefore, we suggest the need to implement a new standard for prescribing rehabilitation in a personalized manner while considering stroke severity and disability, as well as efficient distribution of medical resources for the long-term care of patients with stroke. In particular, rehabilitation prescriptions should focus on the return of patients with mild disabilities to society, and patients with severe disabilities requiring standard treatment should be allowed to receive appropriate treatment for longer periods in rehabilitation wards/hospitals. In addition, it is necessary to establish an efficient system that meets the need for continuous management of mild stroke survivors, even after home discharge or return to society. Consequently, we believe that this study’s findings could inform the development of rehabilitation treatment guidelines and the provision of customized rehabilitation treatments to stroke survivors with varying levels of disability.

This study has several limitations. First, some rehabilitation types, such as speech and cognitive therapies, are not covered by the NHIS. Therefore, our special rehabilitation codes cannot accurately reflect these non-NHIS-covered treatments. Second, death in the chronic phase is associated with medication for secondary causes, continuous follow-up, and patient compliance, which were not covered in this study. Third, we could not provide patient-specific information on primary care for patients in the chronic phase. Fourth, although we analyzed all-cause mortality as the endpoint, we could not determine the cause of death because of the limitations of the data used for the study. Likewise, we could not analyze other outcomes, such as serial functional recovery or quality of life other than death, due to the dataset limitations.

## 5. Conclusions

Under the current NHIS system in South Korea, we observed that, unlike the degree of disability, the frequency of acute/subacute rehabilitation treatments was not necessarily associated with the risk of long-term mortality among stroke survivors with mild-to-moderate disabilities. Therefore, we suggest the need to establish customized rehabilitation and long-term treatment plans based on the degree of disability in stroke survivors. Furthermore, medical resources should be efficiently distributed to stroke survivors. In particular, we believe that this study can act as a reference guide for all the abovementioned purposes.

## Figures and Tables

**Table 1:** Description of the Korean National Disability Registration grades of brain lesion 4 to 6.

Grade	Degree of Disability
4	- Walking and most activities of daily living are performed by oneself but occasionally require assistance from others. - Modified Barthel index: 70–80
5	- Walking and most activities of daily living are performed independently without assistance from others, but sometimes they cannot be performed flawlessly. - Modified Barthel index: 81–89
6	- Walking and most activities of daily living are performed independently and perfectly, but sometimes the execution time is slow, or the execution pattern is abnormal. - Modified Barthel index: 90–96

**Table 2:** Patients’ characteristics.

Variables	Number of Rehabilitations ^a^					*p*-Value
	None	1–50	51–200	201–400	>400	
Total, n	254	209	138	76	56	
Age groups, n (%)						0.241
40–49	9 (3.54)	12 (5.74)	5 (3.62)	8 (10.53)	5 (8.93)	
50–59	42 (16.54)	30 (14.35)	36 (26.09)	12 (15.79)	13 (23.21)	
60–69	82 (32.28)	68 (32.54)	43 (31.16)	26 (34.21)	17 (30.36)	
70–79	95 (37.40)	80 (38.28)	45 (32.61)	25 (32.89)	18 (32.14)	
≥80	26 (10.24)	19 (9.09)	9 (6.52)	5 (6.58)	3 (5.36)	
Men, n (%)	146 (57.48)	122 (58.37)	85 (61.59)	45 (59.21)	28 (50.00)	0.683
Subtypes, n (%)						0.021
SAH	5 (1.97)	5 (2.39)	1 (0.72)	0 (0.00)	1 (1.79)	
ICH	29 (11.42)	27 (12.92)	32 (23.19)	20 (26.32)	7 (12.50)	
Ischemia	213 (83.86)	175 (83.73)	104 (75.36)	55 (72.37)	48 (85.71)	
Unspecified	7 (2.76)	2 (0.96)	1 (0.72)	1 (1.32)	0 (0.00)	
NHIP levels, n (%)						0.592
Medical aid	19 (7.48)	13 (6.22)	8 (5.80)	6 (7.89)	6 (10.71)	
First quartile	48 (18.90)	38 (18.18)	17 (12.32)	9 (11.84)	11 (19.64)	
Second quartile	44 (17.32)	32 (15.31)	16 (11.59)	11 (14.47)	8 (14.92)	
Third quartile	60 (23.62)	51 (24.40)	41 (29.71)	15 (19.74)	14 (25.00)	
Fourth quartile	83 (32.68)	75 (35.89)	56 (40.58)	35 (46.05)	17 (30.36)	
Residential areas, n (%)						0.037
Capital	42 (16.54)	44 (21.05)	34 (24.64)	19 (25.00)	12 (21.43)	
Metropolitan	51 (20.08)	41 (19.62)	40 (28.99)	19 (25.00)	14 (25.00)	
City	109 (42.91)	88 (42.11)	51 (36.96)	29 (38.16)	27 (48.21)	
County	52 (20.47)	36 (17.22)	13 (9.42)	9 (11.84)	3 (5.36)	
HTN, n (%)	216 (85.04)	169 (80.86)	116 (84.06)	59 (77.63)	43 (76.79)	0.380
Diabetes, n (%)	129 (50.79)	96 (45.93)	73 (52.90)	34 (44.74)	21 (37.50)	0.261
DL, n (%)	128 (50.39)	97 (46.41)	63 (45.65)	34 (44.74)	37 (66.07)	0.076
IHD, n (%)	35 (13.78)	12 (5.74)	12 (8.70)	8 (10.53)	5 (8.83)	0.069
AF, n (%)	25 (9.84)	15 (7.18)	13 (9.42)	8 (10.53)	9 (16.07)	0.376
CKD, n (%)	6 (2.36)	4 (1.91)	2 (1.45)	1 (1.32)	1 (1.79)	0.965
Disability grades, n (%)						<0.001
Grade 6	78 (30.71)	51 (24.40)	22 (15.94)	11 (14.47)	4 (7.14)	
Grade 5	67 (26.38)	72 (34.45)	42 (30.43)	17 (22.37)	17 (30.36)	
Grade 4	109 (42.91)	86 (41.15)	74 (53.62)	48 (63.16)	35 (62.50)	

Abbreviations: SAH, subarachnoid hemorrhage; ICH, intracranial hemorrhage; NHIP, national health insurance premium; HTN, hypertension; DL, dyslipidemia; IHD, ischemic heart disease; AF, atrial flutter/fibrillation; CKD, chronic kidney disease. ^a^ From onset to 24 months (claim code: MM105).

**Table 3:** Cox-proportional hazards model for long-term mortality in patients with stroke and mild-to-moderate disabilities.

Variables		Adjusted HR	95% CI	*p*-Value
Disability grades	Grade 6	1.00		
	Grade 5	1.20	0.77–1.86	0.420
	Grade 4	1.82	1.23–2.68	0.003
Number of rehabilitations ^a^	None	1.00		
	1–50	0.79	0.56–1.10	0.160
	51–200	0.80	0.54–1.19	0.277
	201–400	0.67	0.39–1.15	0.143
	>400	0.71	0.39–1.31	0.279
Sex	Men	1.82	1.36–2.44	<0.001
Age groups	40–49	1.00		
	50–59	1.15	0.37–3.60	0.814
	60–69	3.15	1.08–9.17	0.036
	70–79	5.80	2.01–16.76	0.001
	≥80	11.40	3.74–34.77	<0.001
Subtypes	SAH	1.00		
	ICH	0.93	0.21–4.09	0.925
	Ischemia	1.03	0.26–4.35	0.966
	Unspecified	0.25	0.22–2.89	0.268
Co-morbidities	HTN	0.83	0.58–1.19	0.300
	Diabetes	1.14	0.86–1.51	0.374
	DL	0.96	0.72–1.28	0.797
	IHD	0.92	0.58–1.45	0.712
	AF	1.23	0.79–1.92	0.367
	CKD	2.85	1.17–6.96	0.021
NHIP levels	Medical aid	1.00		
	First quartile	1.19	0.64–2.20	0.588
	Second quartile	1.08	0.57–2.07	0.814
	Third quartile	1.20	0.67–2.16	0.540
	Fourth quartile	0.69	0.39–1.23	0.211
Residential Areas	Capital	1.00		
	Metropolitan	1.47	0.95–2.29	0.084
	City	1.17	0.78–1.74	0.456
	County	1.54	0.97–2.44	0.066

Abbreviations: AF, atrial flutter/fibrillation; CI, confidence interval; CKD, chronic kidney disease; DL, dyslipidemia; HTN, hypertension; HR, hazard ratio; ICH, intracranial hemorrhage; IHD, ischemic heart disease; NHIP, National Health Insurance Premium; SAH, subarachnoid hemorrhage. ^a^ rom onset to 24 months (claim code: MM105).

**Table 4:** Cox-proportional hazard models stratified by national disability registration grades.

Disabilities	Number of Rehabilitations ^a^	Adjusted HR ^b^	95% CI	*p*-Value
NDR grade 4	None	1.00		
	1–50	0.64	0.40–1.02	0.059
	51–200	0.46	0.27–0.79	0.005
	201–400	0.50	0.26–0.97	0.040
	>400	0.54	0.25–1.16	0.112
NDR grade 5	None	1.00		
	1–50	0.85	0.39–1.84	0.677
	51–200	1.80	0.83–3.90	0.138
	201–400	0.20	0.02–1.72	0.141
	>400	0.58	0.13–2.73	0.494
NDR grade 6	None	1.00		
	1–50	0.94	0.40–2.20	0.878
	51–200	0.57	0.11–2.86	0.494
	201–400	2.15	0.62–7.43	0.227
	>400	15.00	2.03–111.10	0.008

Abbreviations: CI, confidence interval; HR, hazard ratio; NDR, national disability registration; NHIP, National Health Insurance Premium. ^a^ From onset to 24 months (claim code: MM105). ^b^ Adjusted for sex, age, stroke subtypes, co-morbidities, NHIP levels, and residential areas.

## Data Availability

The dataset used in this study was reviewed and approved by the Korean National Health Insurance Sharing Service (https://nhiss.nhis.or.kr, accessed on 1 May 2023). Only authorized researchers can access the dataset.

## Supplementary Materials

The following supporting information can be downloaded at https://www.mdpi.com/article/10.3390/healthcare11111587/s1. Table S1: National health insurance premium amount by each premium quartile in 2018; Table S2: Definition of comorbid disease in this study; Table S3: Baseline characteristics of patients according to the disability grades.

## References

[1] Statistic Korea (2021). 2020 Population and Housing Census (Register-Based Census). http://kostat.go.kr/.

[2] Yousufuddin M., Young N. (2019). Aging and ischemic stroke. Aging.

[3] Katan M., Luft A. (2018). Global Burden of Stroke. Semin. Neurol..

[4] Kim J., Hwang Y.-H., Kim J.-T., Choi N.-C., Kang S.-Y., Cha J.-K., Ha Y.S., Shin D.-I., Kim S., Lim B.-H. (2014). Establishment of Government-Initiated Comprehensive Stroke Centers for Acute Ischemic Stroke Management in South Korea. Stroke.

[5] Statistic Korea (2021). Causes of Death Statistics in 2020. http://kostat.go.kr/.

[6] Sharma M., Hart R.G., Connolly S.J., Bosch J., Shestakovska O., Ng K.K.H., Catanese L., Keltai K., Aboyans V., Alings M. (2019). Stroke Outcomes in the COMPASS Trial. Circulation.

[7] Singh R.-J., Chen S., Ganesh A., Hill M.D. (2018). Long-term neurological, vascular, and mortality outcomes after stroke. Int. J. Stroke.

[8] Stinear C.M., Lang C.E., Zeiler S., Byblow W.D. (2020). Advances and challenges in stroke rehabilitation. Lancet Neurol..

[9] Stuart M., Ryser C., Levitt A., Beer S., Kesselring J., Chard S., Weinrich M. (2016). Stroke Rehabilitation in Switzerland versus the United States: A Preliminary Comparison. Neurorehabil. Neural. Repair..

[10] Yagi M., Yasunaga H., Matsui H., Morita K., Fushimi K., Fujimoto M., Koyama T., Fujitani J. (2017). Impact of Rehabilitation on Outcomes in Patients With Ischemic Stroke. Stroke.

[11] Hou W.-H., Ni C.-H., Li C.-Y., Tsai P.-S., Lin L.-F., Shen H.-N. (2015). Stroke Rehabilitation and Risk of Mortality: A Population-Based Cohort Study Stratified by Age and Gender. J. Stroke Cerebrovasc. Dis..

[12] Chen C.-M., Yang Y.-H., Chang C.-H., Chen P.-C. (2017). Effects of Transferring to the Rehabilitation Ward on Long-Term Mortality Rate of First-Time Stroke Survivors: A Population-Based Study. Arch. Phys. Med. Rehabil..

[13] Chang K.-C., Hung J.-W., Lee H.-C., Yen C.-L., Wu C.-Y., Yang C.-L., Huang Y.-C., Lin P.-L., Wang H.-H. (2018). Rehabilitation Reduced Readmission and Mortality Risks in Patients With Stroke or Transient Ischemic Attack. Med. Care.

[14] Kim D.Y., Kim Y.-H., Lee J., Chang W.H., Kim M.-W., Pyun S.-B., Yoo W.-K., Ohn S.H., Park K.D., Oh B.-M. (2017). Clinical practice guideline for stroke rehabilitation in Korea 2016. Brain Neurorehabil..

[15] Kang J.H., Bae H.J., Choi Y.A., Lee S.H., Shin H.I. (2016). Length of hospital stay after stroke: A Korean nationwide study. Ann. Rehabil. Med..

[16] Chang W.K., Kim W.S., Sohn M.K., Jee S., Shin Y.I., Ko S.H., Ock M., Kim H.J., Paik N.J. (2021). Korean model for post-acute comprehensive rehabilitation (KOMPACT): The study protocol for a pragmatic multicenter randomized controlled study on early supported discharge. Front. Neurol..

[17] Chang W.H., Sohn M.K., Lee J., Kim D.Y., Lee S.G., Shin Y.I., Oh G.J., Lee Y.S., Joo M.C., Han E.Y. (2017). Long-term functional outcomes of patients with very mild stroke: Does a NIHSS score of 0 mean no disability? An interim analysis of the KOSCO study. Disabil. Rehabil..

[18] Teasell R., Foley N., Salter K., Bhogal S., Jutai J., Speechley M. (2009). Evidence-based review of stroke rehabilitation: Executive summary, 12th edition. Top Stroke Rehabil..

[19] Park D., Son K.J., Kim H.S. (2023). Chronic phase survival rate in stroke patients with severe functional limitations according to the frequency of rehabilitation treatment. Arch. Phys. Med. Rehabil..

[20] Boysen G., Marott J.L., Grønbæk M., Hassanpour H., Truelsen T. (2009). Long-Term Survival after Stroke: 30 Years of Follow-Up in a Cohort, the Copenhagen City Heart Study. Neuroepidemiology.

[21] Lee J., Lee J.S., Park S.H., Shin S.A., Kim K. (2017). Cohort Profile: The National Health Insurance Service-National Sample Cohort (NHIS-NSC), South Korea. Int. J. Epidemiol..

[22] Hsieh C.-Y., Huang H.-C., Wu D.P., Li C.-Y., Chiu M.-J., Sung S.-F. (2018). Effect of Rehabilitation Intensity on Mortality Risk After Stroke. Arch. Phys. Med. Rehabil..

[23] Cheng Y.-Y., Shu J.-H., Hsu H.-C., Liang Y., Chang S.-T., Kao C.-L., Leu H.-B. (2017). The Impact of Rehabilitation Frequencies in the First Year after Stroke on the Risk of Recurrent Stroke and Mortality. J. Stroke Cerebrovasc. Dis..

[24] Kwakkel G., Wagenaar R.C., Koelman T.W., Lankhorst G.J., Koetsier J.C. (1997). Effects of Intensity of Rehabilitation After Stroke. Stroke.

[25] Gattringer T., Posekany A., Niederkorn K., Knoflach M., Poltrum B., Mutzenbach S., Haring H.-P., Ferrari J., Lang W., Willeit J. (2019). Predicting Early Mortality of Acute Ischemic Stroke. Stroke.

[26] Wong K.S. (1999). Risk Factors for Early Death in Acute Ischemic Stroke and Intracerebral Hemorrhage. Stroke.

[27] Cumming T.B., Marshall R.S., Lazar R.M. (2012). Stroke, Cognitive Deficits, and Rehabilitation: Still an Incomplete Picture. Int. J. Stroke.

[28] Cramer S.C., Wolf S.L., Adams H.P., Chen D., Dromerick A.W., Dunning K., Ellerbe C., Grande A., Janis S., Lansberg M.G. (2017). Stroke Recovery and Rehabilitation Research. Stroke.

[29] Martinez-Majander N., Ntaios G., Liu Y.Y., Ylikotila P., Joensuu H., Saarinen J., Perera K.S., Marti-Fabregas J., Chamorro A., Rudilosso S. (2020). Rivaroxaban versus aspirin for secondary prevention of ischaemic stroke in patients with cancer: A subgroup analysis of the NAVIGATE ESUS randomized trial. Eur. J. Neurol..

[30] George M.G. (2020). Risk Factors for Ischemic Stroke in Younger Adults: A Focused Update. Stroke.

[31] Putaala J. (2016). Ischemic stroke in the young: Current perspectives on incidence, risk factors, and cardiovascular prognosis. Eur. Stroke J..

[32] Langhorne P., Bernhardt J., Kwakkel G. (2011). Stroke rehabilitation. Lancet.

[33] Dhamoon M.S., Moon Y.P., Paik M.C., Boden-Albala B., Rundek T., Sacco R.L., Elkind M.S. (2009). Long-term functional recovery after first ischemic stroke: The Northern Manhattan Study. Stroke.

[34] Kim W.S., Bae H.J., Lee H.H., Shin H.I. (2018). Status of rehabilitation after ischemic stroke: A Korean nationwide study. Ann. Rehabil. Med..

