# Supplementary material for: Effect of the Frequency of Rehabilitation Treatments on the Long-Term Mortality of Stroke Survivors with Mild-to-Moderate Disabilities under the Korean National Health Insurance Service System
Source: Healthcare (Basel). 2023 May 29;11(11):1587. doi: 10.3390/healthcare11111587 (PMC10252314; doi:10.3390/healthcare11111587)
Supplement: Supplementary file 1 [file healthcare-11-01587-s001.zip › healthcare-2405796-supplementary.pdf]

Table S1. National health insurance premium amount by each premium quartile in 2018.

Groups	NHIP quartile	Mean monthly insurance premium (KRW)
Medical-aid		0
	1	3,660
	2	9,890
1st NHIP group	3	40,560
	4	46,800
	5	49,920
	6	53,040
	7	56,160
2nd NHIP group	8	62,400
	9	68,640
	10	74,880
	11	78,000
	12	84,240
3rd NHIP group	13	93,600
	14	109,200
	15	124,800
	16	140,400
	17	156,000
4th NHIP group	18	187,200
	19	218,400
	20	312,000

Abbreviations: KRW: Korea Won; NHIP, national health insurance premium

Table S2. Definition of comorbid disease in this study

Comorbidities	ICD-10 codes for the confirmation
HTN	I10, I11, I12, I13, I15
Diabetes	E10, E11, E12, E13, E14
DL	E78
IHD	I25
AF	I48
CKD	N18

Abbreviations: AF, atrial flutter/fibrillation; CKD, chronic kidney disease; DL, dyslipidemia; HTN, hypertension; ICD, International Classification of Disease; IHD, ischemic heart disease.

Table S3. Baseline characteristics of patients according to the disability grades.

	Disability grades			*P*-value
	**Grade 6**	**Grade 5**	**Grade 4**	
Total, n	166	215	352	
Age groups, n (%)				0.009
40–49	10 (6.02)	17 (7.91)	12 (3.41)	
50–59	40 (24.10)	40 (18.60)	53 (15.06)	
60–69	57 (34.34)	72 (33.49)	107 (30.40)	
70–79	47 (28.31)	74 (34.42)	142 (40.34)	
≥ 80	12 (7.23)	12 (5.58)	38 (10.80)	
Male, n (%)	106 (63.86)	126 (58.60)	194 (55.11)	0.168
Subtypes, n (%)				0.591
SAH	3 (1.81)	5 (2.33)	4 (1.14)	
ICH	24 (14.46)	38 (17.67)	53 (15.06)	
Ischemia	135 (81.33)	171 (79.53)	289 (82.10)	
Unspecified	4 (2.41)	1 (0.47)	6 (1.70)	
NHIP levels, n (%)				0.563
Medical-aid	13 (7.83)	14 (6.51)	25 (7.10)	
1st-quartile	35 (21.08)	37 (17.21)	51 (14.49)	
2nd-quartile	25 (15.06)	33 (15.35)	53 (15.06)	
3rd-quartile	41 (24.70)	58 (26.98)	82 (23.30)	
4th-quartile	52 (31.33)	73 (33.95)	141 (40.06)	
Residential areas, n (%)				0.598
Capital	30 (18.07)	50 (23.26)	71 (20.17)	
Metropolitan	36 (21.69)	49 (22.79)	80 (22.73)	
City	79 (47.59)	82 (38.14)	143 (40.63)	
County	21 (12.65)	34 (15.81)	58 (16.48)	
HTN, n (%)	135 (81.33)	178 (82.79)	290 (82.39)	0.930
Diabetes, n (%)	88 (53.01)	100 (46.51)	165 (46.88)	0.361
DL, n (%)	91 (54.82)	101 (46.98)	167 (42.44)	0.230
IHD, n (%)	18 (10.84)	20 (9.3)	34 (9.66)	0.873
AF, n (%)	19 (11.45)	22 (10.23)	29 (8.24)	0.471
CKD, n (%)	3 (1.81)	3 (1.40)	8 (2.27)	0.756

Number of rehabilitation†, n (%)				<0.001
None	78 (46.99)	67 (31.16)	109 (30.97)	
1–50	51 (30.72)	72 (33.49)	86 (24.43)	
51–200	22 (13.25)	42 (19.53)	74 (21.02)	
201–400	11 (6.63)	17 (7.91)	48 (13.64)	
> 400	4 (2.41)	17 (7.91)	35 (9.94)	

†from onset to 24 months (claim code: MM105)

Abbreviations: AF, atrial flutter/fibrillation; CKD, chronic kidney disease; DL, dyslipidemia; HTN, hypertension; ICH, intracranial hemorrhage; IHD, ischemic heart disease; NHIP, national health insurance premium; SAH, subarachnoid hemorrhage.
